# Pulmonary rehabilitation in patients with mustard gas lung disease: a study protocol for a randomized controlled trial

**DOI:** 10.1186/s13063-019-3180-3

**Published:** 2019-02-14

**Authors:** Mohamad Reza Sedighi Moghadam, Mostafa Ghanei, Klaus Kenn, Nicholas S. Hopkinson

**Affiliations:** 1 Janbazan Medical and Engineering Research Centre (JMERC), No 17, Farokh Street, Moghaddas Ardabili Street, Tehran, Iran; 2 Chemical Injuries Center, Systems Biology and Poisonings Institute, Baqiyatallah University of Medical Sciences, Molla Sadra Street, Tehran, Iran; 3 Schön Klinik Berchtesgadener Land, Malterhöh 1, 83471 Schönau am Königssee, Germany; 4 National Heart and Lung Institute, Imperial College London Faculty of Medicine, Royal Brompton Campus, London, SW3 6NP UK

**Keywords:** Pulmonary rehabilitation, Chemical warfare, Mustard gas, Randomized controlled trial

## Abstract

**Background:**

More than 60,000 people have health problems due to chemical weapons exposure during the Iran–Iraq war. Respiratory consequences of mustard gas exposure are common and disabling; medical interventions have limited effect. Patients complain of cough, sputum, breathlessness and exercise limitation. We hypothesized that patients with this condition would benefit from pulmonary rehabilitation.

**Methods:**

We outline the protocol for an assessor-blind, two-armed, parallel-design randomized controlled clinical trial (IRCT2016051127848N1). Sixty patients with respiratory disease due to documented sulfur mustard gas exposure will be randomized to either take part in a 6-week pulmonary rehabilitation programme or receive usual care. Inclusion criteria include forced expiratory volume in 1 second < 80% predicted and Medical Research Council dyspnoea score ≥ 3. The primary endpoint will be the change in cycle endurance time at 70% baseline exercise capacity at 6 weeks. Lung function, physical activity, the strength and endurance of the quadriceps muscle, and quality of life will also be compared. Outcomes will be assessed at 6 weeks and 12 months. Health care utilization will also be assessed.

**Discussion:**

If the study confirms that rehabilitation is effective for patients with mustard gas lung disease this should prompt provision of the intervention to this patient group.

**Trial registration:**

Iranian Registry of Clinical Trials, IRCT2016051127848N1. Registered on 24 May 2016.

**Electronic supplementary material:**

The online version of this article (10.1186/s13063-019-3180-3) contains supplementary material, which is available to authorized users.

## Background

There are more than 60,000 victims of chemical weapons used against Iranian combatants and civilians during the Iran–Iraq war in the 1980s [1]. Mustard gas has well-described respiratory complications, including tracheal stenosis, tracheomalacia, bronchiectasis and constrictive bronchiolitis [2–4]. Patients experience cough, sputum and breathlessness associated with limitation of their exercise capacity and difficulties with day-to-day activities. Fatigue, anxiety and depression are also common [5, 6]. Clinical symptoms and quality of life are in some studies only poorly correlated with pulmonary function [7]. The clinical picture is thus similar to that observed in chronic obstructive pulmonary disease (COPD) [8].

Exercise capacity and breathlessness in lung disease can be influenced by a combination of cardiorespiratory impairment, skeletal muscle dysfunction and changes to the threshold at which symptoms are tolerated [9, 10]. Breathlessness, like pain, can be modified by past experience and expectations, and there is evidence of central adaptations in people with lung disease [10, 11]. A vicious cycle of breathlessness, immobility and deconditioning can worsen the patients’ condition and make them more susceptible to the development of physical inactivity-related multimorbidity including hypertension, diabetes and osteoporosis. Most of the people exposed to mustard gas in the 1980s are now in their 50s or older [1]. A sustainable health system needs to prevent the development of future health problems—physical activity interventions have a key role in this [12].

Pulmonary rehabilitation (PR) is a holistic approach for people limited by lung disease, defined as ‘a comprehensive intervention based on a thorough patient assessment followed by patient-tailored therapies that include, but are not limited to, exercise training, education on your lung disease or condition and how to manage it, and behaviour change, designed to improve the physical and psychological condition of people with chronic respiratory disease and to promote the long-term adherence to health-enhancing behaviours’ [13]. There is an extensive evidence base for PR in COPD patients [14] and a growing body of research in other lung diseases [13]. PR can improve muscle strength, exercise capacity, health status and symptoms of anxiety and depression as well as reducing hospital readmission rates [13]. It has been shown to alter breathlessness perception [10] and also boosts COPD patients’ understanding of their condition and ability to self-manage.

Skeletal muscle impairment is a common feature of COPD and other lung diseases such as pulmonary fibrosis [15]. It can occur early in COPD; weakness is present in about 30% of patients and is associated with reduced levels of physical activity [16]. It directly influences exercise performance [17], is associated with poor health status [18] and is an independent predictor of health care utilization [19] and mortality [20]. Musculoskeletal disorders are also highly prevalent in COPD [21].

A marked increase in susceptibility to fatigue is also observed in COPD, with a more rapid decline in performance during continuous or repeated bouts of exercise [22] or with non-volitional testing [23]. This is associated with a fibre type shift [24], reduced oxidative capacity [23, 24] and increased skeletal muscle adiposity [25]. This fibre type shift is itself independently associated with increased mortality risk [26].

Although the disease shares many clinical features with COPD, few data exist on skeletal muscle parameters in patients with mustard gas lung disease. It is likely that similar processes are in place. In spite of inhaled and other therapies, the clinical condition of chemical warfare victims is progressing with time and as they age [3]. We hypothesize that, as is the case in COPD patients, pulmonary rehabilitation will improve the health of this patient group. To address this, we will conduct a randomized controlled trial to address the following key questions: does PR improve exercise capacity; does PR improve health status; does PR influence daily physical activity levels; does PR increase quadriceps strength; does PR increase quadriceps cross-sectional area; and does PR reduce health resource utilization?. In order to improve the completeness of trial protocols, we used SPIRIT 2013 Checklist (Additional file 1).

## Methods

### Study design

**Fig. 1:**
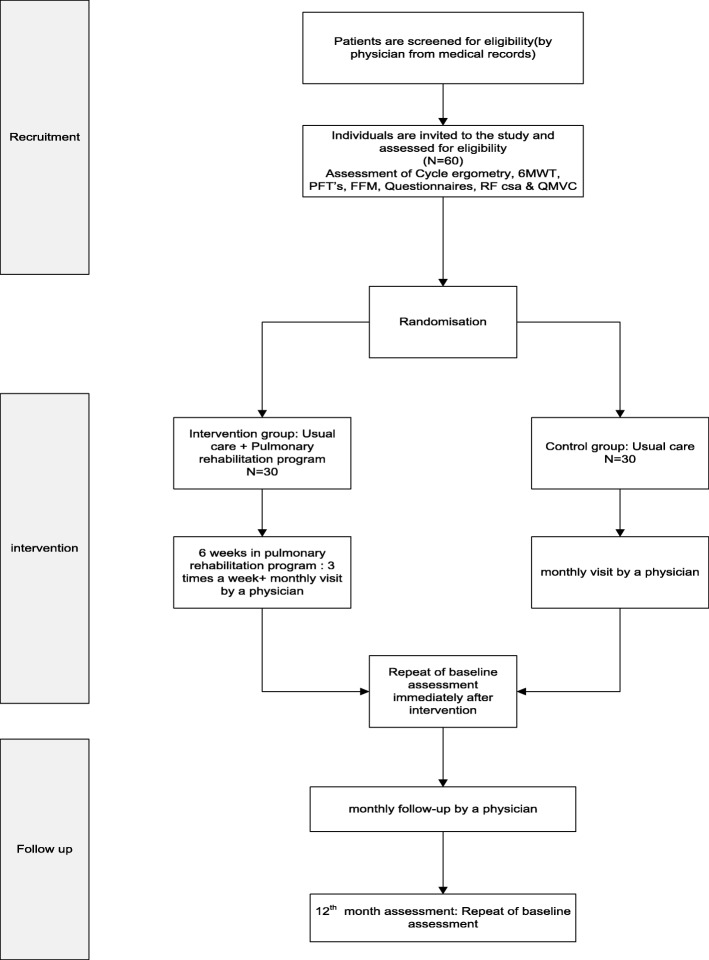
Flow diagram of recruitment, intervention, follow-ups and assessment. Procedure of the study evaluating the impact of the pulmonary rehabilitation programme. 6MWT six-minute walk test, FFM fat-free mass, RF csa rectus femoris cross-sectional area, PFTs pulmonary function tests, QMVC quadriceps maximal voluntary contraction

We have designed an assessor-blind, parallel-group, randomized clinical trial comparing PR with usual care to assess the effect of this intervention in mustard lung patients (Fig 1).

### Study setting

The pulmonary rehabilitation, trial activities and other clinical services will be delivered in Tehran’s Khatam-ol-Anbia Hospital. This hospital is a sub-specialized centre equipped with specialized personnel and advanced equipment including a cardiopulmonary rehabilitation hall. It is located in the city centre which makes it easier to access. Moreover, one of the missions of this hospital is to deliver specialized and sub-specialized services to war veterans [27].

### Participants

The participants are chemical warfare victims with contemporaneously documented exposure to mustard gas, residing in Tehran and its suburbs, who have symptomatic lung disease. Inclusion criteria include FEV_1_ < 80% and MRC dyspnoea score ≥ 3 [28].

Exclusion criteria are: any type of debilitating clinical condition that prevents the patient from participating in the rehabilitation programme, such as arthritis; any type of clinical condition that will endanger the patient during the physical exercises, such as uncontrolled cardiac diseases; the presence of other non-chemical-related pulmonary diseases, like asthma; active malignancy; and severe cognitive disorder and psychiatric disease that is associated with memory disorder.

### Interventions

#### Intervention group

In addition to the usual treatment, the intervention group will receive pulmonary rehabilitation services. The pulmonary rehabilitation intervention will include a combination of physical exercises, an educational programme and psychosocial support. The physical exercises will include endurance and strength components, tailored to each individual’s condition and characteristics, prescribed according to the following protocol. Both the upper and lower limbs will be exercised, although with greater emphasis on the legs. For more details of the intervention protocol, please see Additional file 2.

#### Usual care

**Fig. 2:**
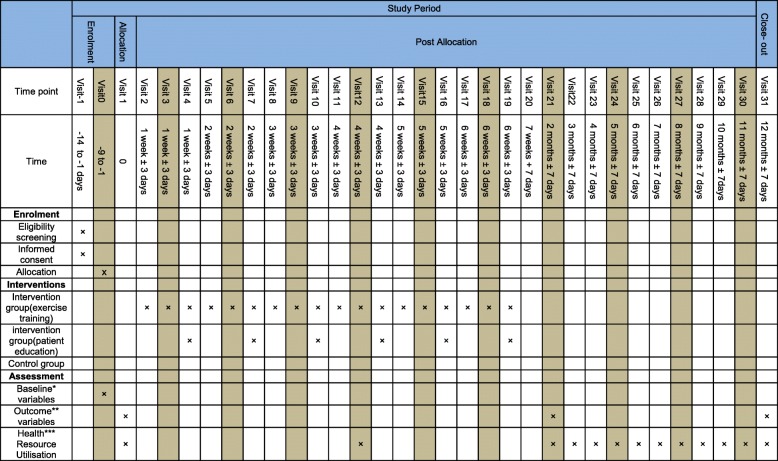
Steps taken at each visit. *Demographics and past medical history. **Dyspnoea scale, fatigue scale, body plethysmography parameters, diffusing capacity/transfer factor of the lung for carbon monoxide parameters, rectus femoris cross-sectional area, incremental cycle exercise test parameters, constant work rate cycle endurance test parameters, isokinetic quadriceps muscle function test parameters, body composition parameters, six-minute walk test parameters, Hospital Anxiety and Depression Scale parameters, St. George’s Respiratory Questionnaire parameters, blood test parameters and daily physical activity parameters. ***Number of exacerbations of the disease, hospitalizations and medications

The control group will continue on their usual medication with management determined by their clinical team. They will receive standard advice about the importance of physical activity and be encouraged to exercise regularly. The participants in both groups will visit 12 times, at 1-month intervals (Fig. 2).

### Outcomes

#### Outcome assessment

Before starting the outcome assessment, assessors will be trained in and familiar with the assessment protocol and instruments. They will be blind to the study participants’ group allocations. Outcomes will be measured at baseline prior to the intervention, after 6 weeks and after 12 months from study entry.

#### Primary outcome

The primary outcome of the study will be the cycle endurance time at 70% of baseline workload, measured at the end of 6 weeks. An increase of > 105 s in the endurance time has been defined as the minimum clinically important difference and will be used as a threshold for responder analysis [29].

A symptom-limited incremental exercise test (IET) will be used to determine the W_max_ of each participant at baseline. After 3 min of acclimatization and 1 min of unloaded cycling, the workload is increased by 10 W/min until the patient can no longer bear heavier loads. The highest workload that each individual can complete during 60 s of pedalling will be defined as the W_max_ [30]. A practice constant work rate test will be performed approximately 1 h after the IET at 70% maximum workload. Three minutes of unloaded cycling (0 W) will be given to allow for respiratory adaptation and warm up [31]. At the next session, a few days later, the constant workload test will be repeated and the outcome of this taken as the baseline measure of the endurance time [30].

### Secondary outcomes/clinical and behavioural

#### Pulmonary function

Spirometry, body plethysmography and gas transfer (DLco) will be measured in accordance with international guidelines [31–33].

#### Quadriceps strength and endurance

Participants will perform 30 repetitions of a maximum voluntary contraction with an angular velocity of 90° per second using the Isokinetic Test Rehabilitation System (MPS21) in an upright sitting position, with an angle of 90° at the thigh joint [34]. Strength is assessed as the quadriceps muscle peak torque (in Newton-meters). The endurance of the quadriceps muscle is defined as the total work done (Joules) during 30 consecutive muscular contraction repeats [35]. To reduce the effects of learning at the baseline assessment, the test will be performed twice and the best value used.

#### Quadriceps muscle bulk

The rectus femoris cross-sectional area (RFCSA) will be measured with B-mode ultrasonography using an 8-MHz, 5.6-cm linear probe, similar to de Bruin et al.’s method [36]. The probe will be placed perpendicular to the longitudinal axis of the thigh in the superior segment, three-fifths of the distance from the anterior superior iliac spine to the superior patellar border. The highest point of the thigh from the RFCSA can be viewed in one field in all individuals. The other quadriceps muscles cannot be investigated by this method. Imaging will be done when the leg is at rest and in a condition wherein the individual is lying supine. Oblique imaging will be minimized by the operator to achieve the smallest cross-sectional image. The depth of the scan is set to where the femur alignment can be recognized. The contraction–relaxation manoeuvre will be used to delineate the muscle septa before the image is taken. Once the inner echogenic line of the rectus femoris is delineated by a movable cursor on a frozen image, the RFCSA will be calculated using a planimetric technique [16, 37]. This outcome will be assessed three times, at baseline, the 6th week and the 12th month.

#### Six-minute walk distance

The 6MWT is done in accordance with ATS guidelines [38], including a practice walk. The better test will be considered the patient’s baseline assessment. The patients will not do warm-ups before the test and will rest for at least 10 min before the test is begun. Any contraindications to the test will be sought. The blood pressure, heart rate and SpO_2_, as well as dyspnoea and leg fatigue with the Borg scale, will be recorded. Patients will receive standard instructions on going the greatest distance during 6 min. They will be monitored throughout the test for SpO_2_. If a pause is required due to dyspnoea or fatigue, the patient will be allowed to rest. However, the chronometer will not be stopped and the numbers and durations of stops will be noted.

At the end of the 6 min, in addition to registering the distance walked, the SpO_2_ and heart rate will be measured, and the level of dyspnoea and leg fatigue will be measured with the Borg scale.

#### Daily physical activity

Daily physical activity will be measured with an accelerometer (triaxial pedometer PD724; Tanita, Tokyo, Japan). The patient will use the pedometer according to instructions for a week at three stages: a week before they start the programme at baseline, the 6th week and the 12th month. Participants will be asked to bring the pedometer after a week. The Tanita PD724 pedometer displays a cumulative step count for each day and also retains the step counts from the preceding 7 days in its memory, allowing the average step count for the preceding week to be evaluated [39]. The first and last days of handing over the pedometer will not be included in the estimations due to the possibility of bias.

#### Quality of life

St. George’s Respiratory Questionnaire (SGRQ) is a standard self-administered questionnaire that has been specifically designed to assess QOL in patients with chronic respiratory diseases [40]. The Persian version has also been standardized and domesticized, and can be used to evaluate QOL in patients with chronic pulmonary diseases [41]. This tool consists of three sections: symptom, activity and impact. In the first section, the symptoms are assessed, and items such as the frequency of cough, sputum production, wheeze, breathlessness and duration and frequency of attacks of breathlessness or wheeze are evaluated. Activity and impact are examined in the next sections and include the activities that bring about breathlessness, or the activities that are limited due to breathlessness. Moreover, the impact of respiratory problems on life and activities such as employment, daily life disorders and so forth are evaluated [41].

#### Anxiety and depression

The HADS questionnaire is a well-known tool to screen for anxiety and depression, and can detect the impact of therapeutic interventions. Studies conducted on the validity of this questionnaire in detecting the symptoms and causes of anxiety and depression and assessing their severity have indicated its high accuracy and high validity. The Persian version has also been standardized and domesticized, and can be used [42]. This tool contains seven questions for assessing anxiety and seven questions for assessing depression, scored from 0 to 3. Thus, each individual can gain 0–21 for anxiety or depression [43]. Based on the results of a systematic review on a number of studies employing this tool, the cut-off point for anxiety or depression is 8/21 [44].

#### Body composition

The participants will be asked to stand barefoot while their height is measured to the nearest 0.5 cm. A single-frequency bioelectrical impedance analyser (BIA) will be used to assess body composition. Height (cm), weight (kg), FFM (kg) and BMI (kg/m^2^) will be measured at baseline, the 6th week and the 12th month.

#### Blood tests

Testosterone, high-sensitivity CRP, IGF-1 and GH will be measured as factors that may be associated with reduced muscle mass. These tests will be measured at baseline, the 6th week and the 12th month.

#### Number of disease exacerbations and hospitalizations

To measure this outcome, the number of exacerbations of disease and cases of hospitalizations due to pulmonary disease during the past year will be asked. An exacerbation is defined as ‘an increase in respiratory signs and symptoms leading to change in type and/or rate of medicine intake’. This outcome will be assessed based on recall and clinical records at baseline, the 6th week and the 12th month. Heath resource utilization will be assessed from a health service perspective. Data will be captured from central clinical records and include hospital visits, medication and medical procedures performed. These will be related to standard costs to allow for an estimate of the health economic impact of the intervention to be assessed.

### Randomization

After selecting the participants according to the inclusion and exclusion criteria, baseline assessments will be performed. The participants will then be randomized into control or intervention groups. A random sequence will be determined from the randomization website (http://www.jerrydallal.com/random/permute.htm) in blocks of four. Randomization will be performed by a technician who is blind to the title of each group. In order to protect confidentiality in all stages of the trial, each participant will be assigned a unique identification code.

### Recruitment and consent

Chemical warfare victims residing in Tehran and its suburbs will be identified from the administrative database for this condition. Those with a history of exposure to mustard gas will be invited to participate in the study. The patients will undergo specialized examinations and baseline assessments. Patients with the inclusion criteria will read the informed consent form (Additional file 3), previously provided by study supervisor, and receive further explanation in the case of any confusion regarding the contents. If the patient needs more time to think or to consult their family members they will be given a week to decide.

During the study, the patient will be excluded from the study if any of the following occurs: any of the exclusion criteria emerge in the patient; the patient is affected with any disease or medical condition that will make it difficult for them to continue participating in the study; the patient wishes to withdraw from the study for personal reasons; emigration; or death. Compliance with PR is defined as missing fewer than four sessions and a per-protocol analysis as well as intention to treat will be carried out. During the trial, patients can receive their care but they will not be allowed to undergo any interventional study.

### Follow-up

The participants of both groups will be visited 12 times, at 1-month intervals (Fig. 1). At the ‘zero visit’, all individuals who are most likely eligible (based on their medical records) to enrol in the study will be invited. The participants will then be carefully examined by specialists and assessed for presenting the inclusion or exclusion criteria.

### Data management

Patient data will be collected from their medical records, questionnaires completed by the questioner, test reports, documented physician comments, researchers’ observations and patient interview. A technician will enter the data from the ‘Case Report Form’ (CRF) into SPSS statistical software. To ensure the accuracy of data entry, double data entry will take place at every stage of data gathering.

The Coordinating Team, Steering Committee, Endpoint Adjudication Committee, Data Management Team and Data Monitoring Committee are responsible for the implementation, control and monitoring of the correct implementation of the study.

### Sample size and statistical analysis

Assuming a 105-s improvement in the time limit in the treatment arm and a 20-s improvement in controls with an SD of 100, 90% power and a significance level of 5% requires 60 patients to complete the study.

The data will be cleaned and assessed for outliers and missing variables. Outcomes will be compared using ANOVA adjusted for baseline parameters. The data will be analysed by *t* test, chi-square test and, if required, non-parametric tests and with Excel 2010 and SPSS Statistics 20 software at a level of significance of 0.05.

## Discussion

This study using a controlled randomized clinical trial will investigate the impact of a PR programme on people with mustard gas lung disease. If the programme proves efficacious, pulmonary rehabilitation centres can be established and expanded across the country/countries that have been victims of chemical attacks.

Among the limitations of this study are the following: participation in the rehabilitation centre exercises for 6 weeks, three times a week might be difficult for those participants who are employed; and the length of the project (being 1 year long) raises the risk of participant drop-out.

Some of the strengths of this study are as follows: this is the first PR protocol that is being conducted for sulfur mustard victims; the emphasis on the use of objective and safe methods in assessing patients, instead of subjective methods, is an additional strength of this study; and the results of this study can be used in other regions that have also been victims of mustard gas exposure.

## Trial status

The enrolment of the first participant to the trial was performed on 22 November 2017 and the enrolment was completed in August 2018. The participants were randomized and allocated and last patient last visit will be in August 2019.

## Additional files

Additional file 1: SPIRIT 2013 Checklist: Recommended items to address in a clinical trial protocol and related documents (DOCX 51 kb)

Additional file 2: Intervention protocol. (DOCX 31 kb)

Additional file 3: Participation in the Pulmonary Rehabilitation Project (Persian version). (DOCX 23 kb)

## Abbreviations

6MWT: Six-minute walk test
BIA: Bioelectrical impedance analyser
BMI: Body mass index
COPD: Chronic obstructive pulmonary disease
CRF: Case Report Form
CRP: C-reactive protein
DLco: Diffusing capacity/transfer factor of the lung for carbon monoxide
EAC: Endpoint Adjudication Committee
FEV1: Forced expiratory volume in 1 second
FFM: Fat-free mass
GH: Growth hormone
HADS: Hospital Anxiety and Depression Scale
IET: Incremental exercise test
IGF-1: Insulin-like growth factor 1
MRC: Medical Research Council
PR: Pulmonary rehabilitation
QOL: Quality of life
RFCSA: Rectus femoris cross-sectional area
SGRQ: St. George’s Respiratory Questionnaire
SpO_2_: Saturation of peripheral oxygen
W_max_: Maximum workload
