# Supplementary material for: Pulmonary rehabilitation in patients with mustard gas lung disease: a study protocol for a randomized controlled trial
Source: Trials. 2019 Feb 14;20:132. doi: 10.1186/s13063-019-3180-3 (PMC6376791; doi:10.1186/s13063-019-3180-3)
Supplement: Supplementary file 2 — Intervention protocol. (DOCX 31 kb) [file 13063_2019_3180_MOESM2_ESM.docx]

**Additional file 2**

**Training protocol**

Every session will include sixty minutes of exercise, of which 15 minutes will be spent on warming up. All the exercises will be taught to the participants by a physiotherapist, who will supervise their correct performance throughout. A general physician will be present in each session to ensure rapid aid to the participants, if a problem arises. Participants will undergo eighteen sessions (6 weeks, 3 sessions a week).

**Endurance training**

Initially, they will exercise at 70% of the maximal exercise capacity (Wmax) obtained in an initial incremental cycle ergometer exercise test. Once the patient can perform this level of exercise for 20min without intolerable dyspnea or fatigue, the workload will be increased by 10%. On the treadmill, we will begin with 1.1 – 2 miles per hour initially at 0% elevation. The rate of increment will be determined based on the individual’s functional capacity in the six-minute walk test (6MWT). The speed will be increased when the patient can perform the exercise for 20 minutes without intolerable dyspnea or fatigue. At the beginning of each session, a series of stretch exercises will be performed. Patients will also perform warm-up exercises with or without small weights [[1](#_ENREF_1)]. If the patient cannot perform the exercise for 20 minutes, interval training will be considered as a replacement[[2](#_ENREF_2)]. A sprint exercise will be done at 100% Wmax in the form of 30 seconds of cycling and 30 seconds of rest.

**Strength training**

In addition to endurance training, the patients will perform four different strength exercises. Knee flexion involving specially the hamstring muscle, Knee extension affecting mainly the quadriceps muscle, Chest press in seated position affecting mainly the pectoralis major muscle and a combined movement so that shoulder is adducted and arms are flexed affecting mostly latissimus dorsi [[3](#_ENREF_3)]. Initially, patients are requested to perform one set of 10 repetitions at 60% of their one repetition maximum. If it is tolerated it will be gradually increased to three sets of 10 repetitions. The weight will be added by 5 lbs whenever the patient can perform the sets of three without difficulty [[4](#_ENREF_4)].

**Patient Education**

In the educational section of the rehabilitation intervention, an educational session will be held once a week to promote knowledge and modify individuals’ attitude toward disease and ways of controlling it. Moreover, the skills required for self-management will be developed. The educational program will include the following topics, and each topic will be presented by an appropriate specialist: introduction to the respiratory system, pulmonary effects of sulfur mustard, basis for pulmonary rehabilitation, the benefits of exercise, energy saving strategies, managing stress, nutrition in pulmonary disease, controlling dyspnoea, appropriate use of medications, quitting smoking.

**Psychosocial Support**

In addition to the abovementioned educational programs, four group therapy sessions will be held by a psychologist to improve participants’ attitudes to disease control and to raise self-efficiency to promote and improve quality of life. To improve psychosocial support, the following arrangements will be taken into consideration: introducing the patients to the chemical warfare victims’ NGO, with an eye toward forming a stronger social network between the victims and the NGO members; participation in recreational and educational group programs (with the family); home visits by a physician monthly.

1. Mador MJ, Deniz O, Aggarwal A, Shaffer M, Kufel TJ, Spengler CM: **Effect of respiratory muscle endurance training in patients with COPD undergoing pulmonary rehabilitation.** *CHEST Journal* 2005, **128:**1216-1224.

2. Zainuldin R, Mackey MG, Alison JA: **Optimal intensity and type of leg exercise training for people with chronic obstructive pulmonary disease.** *Cochrane Database of Systematic Reviews* 2011.

3. Bernard S, Whittom F, LeBLANC P, Jobin J, Belleau R, Berube C, Carrier G, Maltais F: **Aerobic and strength training in patients with chronic obstructive pulmonary disease.** *American Journal of Respiratory and Critical Care Medicine* 1999, **159:**896-901.

4. Mador MJ, Bozkanat E, Aggarwal A, Shaffer M, Kufel TJ: **Endurance and strength training in patients with COPD.** *CHEST Journal* 2004, **125:**2036-2045.
