# Supplementary material for: Pulmonary rehabilitation in patients with mustard gas lung disease: a study protocol for a randomized controlled trial
Source: Trials. 2019 Feb 14;20:132. doi: 10.1186/s13063-019-3180-3 (PMC6376791; doi:10.1186/s13063-019-3180-3)
Supplement: Supplementary file 3 — Participation in the Pulmonary Rehabilitation Project (Persian version). (DOCX 23 kb) [file 13063_2019_3180_MOESM3_ESM.docx]

**رضايت‌نامه شرکت در طرح توانبخشی ریوی**

**برادر گرامي**

**بدينوسيله ضمن قدرداني از حضرتعالي به منظور ابراز تمايل براي مشاركت در طرح پژوهشي** ارزیابی تاثيرات توانبخشی ریوی بر وضعیت عضلات اسکلتی، وضعیت کارکرد ریوی و کیفیت زندگی در قربانیان جنگ شیمیایی به اطلاع می رساند که این طرح **كه با اهداف زیر به تصويب شوراي علمي** پژوهشکده مهندسی و علوم پزشکی جانبازان **و كميته‌ي اخلاق در پژوهش بنيادشهيد و امورايثارگران رسيده‌است به مدت** یک سال  **به اجرا در خواهد آمد، لذا موارد زير به منظور آگاهي كامل شما از مراحل اجرايي و جزئيات طرح ياد شده ارائه** **مي‌گردد؛**

**هدف كلي طرح:** ارزیابی تاثیر توانبخشی ریوی بر روی کیفیت زندگی، وضعیت عضلات اسکلتی و وضعیت کارکرد ریوی در مصدومین شیمیایی با گاز خردل

**سرپرست طرح:** دکتر مصطفی قانعی

**جامعه‌ي مورد مطالعه:** مصدومین شیمیایی تهران و شهر ری

**توضيح و شرح روش اجرا و گرد آوري داده‌ها:** ما قصد داریم در ابتدا توسط مطالعه توصیفی مقطعی از طریق معاینه، انجام آزمایشات پاراکلینیکی و تکمیل پرسشنامه کیفیت زندگی ، وضعیت عضلات اسکلتی و وضعیت کارکرد ریوی را در بیماران ریوی گاز خردل ارزیابی کنیم.سپس بیماران برای یکی از دو روش توانبخشی ریوی و یا مراقبت معمول به طور تصادفی انتخاب شده و به دو گروه تقسیم می شوند. شما ممکن است در یکی از دو گروه قرار داشته باشید. در گروه توانبخشی، توانبخشی ریوی با تمرینات استقامتی هوازی و تمرینات مقاومتی و همچنین آموزش مهارت خود مدیریتی ترکیب خواهد شد. بیماران 3جلسه در هر هفته به مدت 6 هفته در آزمون شرکت می­کنند. در همین مدت گروه دوم درمان معمول خود را دریافت خواهند داشت. کلیه شرکت کنندگان علاوه بر ابتدای برنامه، یکبار در هفته ششم(زمان پایان برنامه توانبخشی ریوی) و یکبار در پایان ماه دوازدهم مورد ارزیابی مجدد قرار خواهند گرفت.

توضيح و شرح خطرات احتمالي:

ورود کلیه شرکت کنندکان در طرح مستلزم ویزیت اولیه توسط تیمی تخصصی متشکل از متخصص ریه، متخصص قلب و متخصص طب فیزیکی /پزشکی ورزشی است تا علاوه بر انتخاب بیمارانی که بتوانند بیشترین بهره را از نتایج طرح ببرند، کلیه افرادی که ممکن است تمرینات فوق برای ایشان هرگونه ضرر احتمالی داشته باشد از برنامه حذف کنند. لذا شرکت کلیه بیماران تایید شده توسط تیم پزشکی در کلیه مراحل این برنامه بدون خطر می باشد. کلیه مراحل ارزیابی بیماران و اجرای برنامه توانبخشی در بیمارستان و در حضور کادر درمانی مرتبط انجام می گردد به همین علت ایمنی بیماران در کلیه مراحل اجرا مد نظر و تحت پایش است.

سرپرست اين طرح تعهد مي‌نمايد كه در صورت بروز هرگونه خطر و يا آسيب مستقيم يا غيرمستقيم بلافاصله شما را در جريان قراردهد و جبران خسارت وارده را انجام دهد.

نتايج حاصل از اين طرح در از طریق طراحی مناسب برنامه توانبخشي ریوی در ارائه خدمات حوزه سلامت به شما مؤثر خواهدبود.

سرپرست طرح و سایر همكاران ، درصورت يافتن هر مشكلي، در هنگام ارزيابي، كه نيازمند انجام مداخلات درماني باشد، شما را به مرجع مربوطه معرفي مي‌نمايد.در این خصوص به معاونت بهداشت و درمان بنیاد استان و مرکز مصدومین شیمیایی اطلاع رسانی خواهد شد تا با هماهنگی مراجع مربوطه، اقدامات درمانی مورد نیاز انجام شود.

شما اجازه داريد در هر زمان كه مايل به ادامه همكاري در طرح نيستيد، با آگاه‌سازي سرپرست طرح، آزادانه از طرح خارج شويد.

اطلاعات دريافتي از شما در طي مطالعه و پس از آن كاملاً محرمانه مانده و بدون اجازه‌ي شما در هيچ مرجعي با ذكر نام شما ارائه نخواهدشد و گزارش‌ها بدون قيد نام و يا گزارش‌هاي كلي و عمومي خواهد بود.

قرارگیری افراد در گروه شاهد موجب محرومیت وی از خدمات ضروری نخواهد شد.

**نام و نام خانوادگي سرپرست طرح : امضاء تاريخ**

**اينجانب فرزند به شماره‌ي شناسنامه: باآگاهي كامل از مطالب فوق و مراحل اجرايي طرح يادشده، رضايت كامل خود را براي مشاركت در آن اعلام مي‌دارم.**

**نام و نام خانوادگي امضاء تاريخ**
